# Elevated Circulating Extracellular Vesicles as Prognostic Biomarkers in Cervical Cancer Progression

**DOI:** 10.3390/biomedicines14071492

**Published:** 2026-06-30

**Authors:** Helder Costa Drumond, Marina Malheiros Araújo Silvestrini, Liliane Martins dos Santos, Fábio Magalhães-Gama, Jorge Gomes Goulart Ferreira, Kassyane Amanda Rodrigues Furtado, Pedro Luiz Lima Bertarini, Matheus de Souza Gomes, Laurence Rodrigues do Amaral, Olindo Assis Martins-Filho, Paulo Guilherme de Oliveira Salles, Letícia Conceição Braga, Andréa Teixeira-Carvalho

**Affiliations:** 1 Grupo Integrado de Pesquisas em Biomarcadores, Instituto René Rachou-Fiocruz, Belo Horizonte 30190-002, Brazil; helder.costa.drumond@gmail.com (H.C.D.); msilvestrini@aluno.fiocruz.br (M.M.A.S.); lilianemartins.bh16@gmail.com (L.M.d.S.); magalhaes.gama.f7@gmail.com (F.M.-G.); olindo.filho@fiocruz.br (O.A.M.-F.); 2 Laboratório de Pesquisa Translacional em Oncologia, Núcleo de Ensino, Pesquisa e Inovação, Instituto Mário Penna, Belo Horizonte 30380-420, Brazil; jorge.ferreira@mariopenna.org.br (J.G.G.F.); kassyanefurtadoacad@gmail.com (K.A.R.F.); paulo.salles@mariopenna.org.br (P.G.d.O.S.); 3 Laboratório de Bioinformática e Análise Molecular, Universidade Federal de Uberlândia (UFU), Campus Patos de Minas, Patos de Minas 38701-002, Brazil; bertarini@ufu.br (P.L.L.B.); matheusgomes@ufu.br (M.d.S.G.); laurence.amaral@gmail.com (L.R.d.A.); 4 Laboratório de Anatomia Patológica, Hospital Luxemburgo, Instituto Mário Penna, Belo Horizonte 30380-420, Brazil

**Keywords:** cervical cancer, liquid biopsy, extracellular vesicles, flow cytometry, biomarkers

## Abstract

**Introduction:** Cervical cancer ranks among the leading cancers in women worldwide, with mortality disproportionately affecting low- and middle-income countries. Traditional diagnostic and prognostic tools have limited sensitivity and specificity, and accessibility challenges, underscoring the need for innovative biomarkers. Recent findings suggest that extracellular vesicles (EVs), released by both tumor and immune cells, may reflect the disease state and serve as minimally invasive biomarkers. This study investigates circulating EVs and their potential role as biomarkers in cervical cancer. **Objective:** To evaluate the levels and cellular origins of circulating EVs in cervical cancer patients across different clinical stages and outcomes, assessing their potential as diagnostic and prognostic biomarkers. **Methods:** In this study, we analyzed 96 cervical cancer patients and 31 healthy controls. Peripheral blood samples were processed to isolate and quantify EVs, followed by immunophenotyping using flow cytometry. Specific markers identified EVs originating from neutrophils, lymphocytes, platelets, and endothelial cells. Comparative analyses were conducted to assess EV profiles in relation to clinical stages and patient outcomes. Statistical significance was set at *p* < 0.05. Machine learning approaches were employed to assess EV performance. **Results:** Circulating EV levels were significantly elevated in cervical cancer patients compared to healthy controls (*p* < 0.01). Immunophenotyping revealed marked increases in EVs derived from neutrophils (CD66^+^, CD16^+^), T lymphocytes (CD3^+^), leukocytes (CD45^+^), platelets (CD41a^+^), and endothelial cells (CD51/CD61^+^), all of which were highly significant (*p* < 0.0001). Monocyte-derived EVs (CD14^+^) and erythrocyte-derived EVs (CD235a^+^) were also significantly elevated (*p* < 0.01 and *p* < 0.001, respectively). When stratified by survival outcomes at 8 months post-treatment, responders exhibited a more pronounced elevation in erythrocyte-derived EVs compared to non-responders and deceased patients (*p* = 0.0002), suggesting a potential association with improved outcomes. Total EV levels were significantly higher in advanced-stage patients (Stages III and IV) than in controls (*p* < 0.05), but not in early-stage patients (Stages I and II). However, EVs derived from specific cell types were significantly increased in both early and advanced stages (all *p* < 0.05), with no significant differences between the stages, indicating a consistent elevation regardless of disease progression. Regarding histopathological grades, total EV levels were significantly elevated in patients with Grade I and Grade III tumors (both *p* < 0.05) but not in those with Grade II tumors. Cell-specific EV elevations were observed across all grades, though with some variations; for instance, monocyte-derived EVs were significantly elevated in Grades I and III (both *p* < 0.05) but not in Grade II. These findings highlight that while elevated EV levels are a hallmark of cervical cancer, specific EV subtypes may have distinct associations with clinical stages, histopathological grades, and patient outcomes. This underscores their potential utility as diagnostic and prognostic biomarkers. **Conclusions:** This study highlights the potential of circulating EVs as non-invasive biomarkers for cervical cancer, with distinct EV profiles associated with disease severity and prognosis. These findings suggest that EV analysis could aid in stratifying patients by risk, enhancing personalized treatment strategies. Future research should explore the molecular cargo within EVs to further elucidate their role in tumor biology and as therapeutic targets.

## 1. Introduction

Cervical cancer remains a significant global health challenge, ranking as the fourth most common malignancy among women worldwide. In 2022, approximately 662,044 cases and 348,709 deaths were reported, underscoring the urgent need for improved diagnostic and prognostic tools [1]. Recent projections by the World Health Organization (WHO) further estimate that, without substantial preventive interventions, the global burden could exceed 700,000 new cases annually by 2030, particularly in low- and middle-income countries (LMICs) [2]. The disease’s progression is often asymptomatic in its early stages, leading to delayed diagnoses and reduced survival rates. Traditional screening methods, such as the Papanicolaou (Pap) smear and high-risk human papillomavirus (hrHPV) testing, have been instrumental in early detection [3]. However, these methods have limitations, including variable sensitivity and specificity, as well as accessibility issues in low-resource settings. Consequently, there is a pressing need to identify novel biomarkers to enhance the efficiency of early detection, prognosis, and therapeutic monitoring of cervical cancer [4].

EVs have garnered significant attention in recent years as potential biomarkers in oncology. EVs are membrane-bound particles ranging from 100 to 1000 nanometers in diameter, released by various cell types into the extracellular environment [5]. They play crucial roles in intercellular communication by transporting bioactive molecules, including proteins, lipids, and nucleic acids, thereby influencing various physiological and pathological processes [6]. The recognition of EVs as active mediators of tumor progression has been reinforced by recent translational reviews summarizing the updated ISEV guidelines and their clinical applicability, particularly highlighting the diagnostic and prognostic value of EVs in liquid biopsy approaches [7]. In the context of cancer, tumor-derived EVs have been implicated in promoting tumor growth and metastasis, and modulating the tumor microenvironment. Their presence in easily accessible body fluids, such as blood, makes them attractive candidates for non-invasive liquid biopsies [8].

Studies have shown that EVs are not only abundant in cancer patients but may also correlate with the disease severity, reflecting tumor-related changes such as cellular turnover and apoptosis [9]. Elevated levels of circulating EVs have been associated with advanced stages of several malignancies, including breast, ovarian, and prostate cancers, where they indicate higher tumor aggressiveness and metastatic potential [10]. More recent clinical studies further demonstrate that the phenotypic composition of EVs, particularly immune- and endothelium-derived subsets, can predict therapeutic response and disease recurrence, underscoring their translational value as precision biomarkers [11]. Such findings underscore the potential of EVs as biomarkers to stratify cervical cancer patients by disease stage and prognosis, thereby enhancing clinical decision-making in both diagnostic and therapeutic contexts. Furthermore, recent research highlights that EVs carry oncogenic proteins, RNA, and other bioactive molecules, which play a role in tumor progression and immune modulation [12]. In cervical cancer, certain microRNAs (miRNAs) encapsulated within EVs have been identified as differentially expressed, suggesting their potential utility as diagnostic and prognostic biomarkers [13]. We hypothesize that EV profiles can be used as tools for cervical cancer progression and prognosis. By investigating the associations between EV levels, cellular origins and cervical cancer progression, this study aims to provide a comprehensive analysis from early to advanced disease stages, thereby offering insights into the prognostic capabilities of EVs specific to cervical cancer. Through a detailed analysis of EV populations derived from both immune and tumor cells, this study examines how EVs correlate with clinical stages and outcomes, setting the stage for their integration into clinical practice as part of a new generation of biomarkers. Understanding the dynamics of EVs in cervical cancer could pave the way for more personalized and effective management strategies, ultimately improving patient outcomes.

## 2. Population, Materials, and Methods

### 2.1. Patient Cohort and Sample Collection

This study was approved by the Research Ethics Committee of the Mario Penna Institute (CAAE: 82703418.8.0000.5121). The research adhered to universal ethical principles and complied with international guidelines, including the Declaration of Helsinki and the International Ethical Guidelines for Biomedical Research Involving Human Subjects (CIOMS), as well as Brazilian ethical regulations (Res. CNS 196/96 and its supplements).

A total of 127 participants were recruited for the study, all of whom were over 18 years old, capable of understanding, and able to provide informed consent. Each participant signed the informed consent form (TCLE). All participants presented with locally advanced cervical cancer and were admitted to the Mario Penna Institute (IMP) in Belo Horizonte, Brazil. Diagnoses were confirmed through colposcopy and histopathological analysis performed at the IMP Pathology Laboratory.

Disease progression was evaluated using the Response Evaluation Criteria in Solid Tumors (RECIST) framework during follow-up, which occurred four to eight months after the completion of chemoradiotherapy. This follow-up interval was established in accordance with institutional clinical protocols and reflects the period during which most early therapeutic responses, local recurrences, and progression events are typically observed in locally advanced cervical cancer. This timeframe allowed for consistent assessment of short-term treatment outcomes while maintaining high patient retention and data completeness.

Patients were excluded from the study if they had previous malignancies, had undergone surgical treatment for cervical cancer, or if chemoradiotherapy was interrupted due to unacceptable toxicity levels. For the collection of circulating EVs, peripheral blood was drawn via venipuncture into 5 mL tubes containing 3.2% sodium citrate (Biocon Diagnósticos^®^, Belo Horizonte, MG, Brazil). The blood was subsequently centrifuged at 800× *g* for 15 min at room temperature to obtain platelet-poor plasma (PPP), which was stored at −80 °C for further analysis.

### 2.2. Isolation and Immunophenotyping of EVs

The PPP samples were thawed in a 37 °C water bath, centrifuged at 8000× *g* for 15 min at 15 °C platelet-free plasma (PFP), and the supernatant was diluted in a citrate buffer solution containing heparin (1 μg/mL) (Vacutainer Blood Collection Tube, BD Medical, Franklin Lakes, NJ, USA). EVs were isolated from plasma samples using a standardized differential centrifugation protocol at 8000× *g* for 90 min at 15 °C. This protocol was previously optimized and validated by our group specifically for compatibility with nanoscale flow cytometric analysis [11,14,15,16].

The sediment containing the EVs was resuspended in commercial Annexin V buffer (25 mM CaCl_2_ solution in 140 mM NaCl and 10 mM HEPES, pH 7.4; BD Biosciences, San Diego, CA, USA). To determine the cellular origin of the EVs, 100 µL of the suspension was transferred to tubes containing 2 µL of monoclonal antibodies conjugated to different fluorochromes, namely, APC-CD45 (clone HI30), PE-CD66b (clone B1.1), PE-CD16 (clone 3G8), PE-CD51/CD61 (clone 23C6), PE-Cy5.5-CD235a (clone GA-R2/HIR2), PE-CD3 (clone HIT3a), PerCP-CD41a (clone HIP8), PerCP-CD14 (clone MEM-15) and FITC-Annexin V (clone DX2 (BD Medical, Franklin Lakes, NJ, USA) followed by incubation for 30 min in the dark, at room temperature.

Approximately 1,000,000 events were acquired with at least 150,000 events within the annexin V-specific region using the flow cytometer Cytoflex S (Beckman-Colter, Brea, CA, USA) equipped with Violet Side Scatter (VSSC) for enhanced nanoscale resolution. Calibration microbeads (Gigamix beads, Stago Co., Marseille, France) with a 100–900 nm range were used to define the analytical gate for EV detection and select the size range of EVs analyzed (Figure S1). Schematic representations of the data analysis strategy are provided for both healthy controls (Figure S2) and the patient cohort (Figure S3). Full analysis data and additional representative plots are available upon request to the corresponding author.

To ensure fluorescence specificity and minimize background signal, acquisition included buffer-only, unstained, and fluorescence-minus-one (FMO) controls for each antibody. All buffers and reagents were filtered through 0.22 μm membranes, (Corning, Kaiserslautern, Germany). and antibodies were ultracentrifuged prior to use to prevent aggregate formation. These optimizations collectively improved the reproducibility and accuracy of EV immunophenotyping.

### 2.3. Statistical Analysis

Statistical analyses were performed using GraphPad Prism v9.0^®^ software (San Diego, CA, USA). To evaluate the data distribution, the Kolmogorov–Smirnov test was performed. For data considered non-parametric, the Mann–Whitney test was performed to compare two groups and the Kruskal–Wallis test to compare three groups, followed by Dunn’s post-hoc test at a 95% confidence level. Signature analyses were performed using the global median across groups. Detailed statistical results are provided in Tables S1–S9.

Performance tests were carried out using MedCalc software version 7.3.0 (Ostend, Belgium; URL: https://www.medcalc.org/, accessed on 1 March 2023) to determine the cut-off value, sensitivity, specificity, and likelihood ratio. The area under the curve (AUC) was determined by GraphPad Prism v9.0^®^ software. For all analyses, values of *p* < 0.05 were considered statistically significant.

### 2.4. Development and Training of CC Classifier Algorithms

Decision trees were generated to classify attributes from EV and clinical data of CC patient datasets using the J48 method and the Waikato Environment for Knowledge Analysis (WEKA) software, version 3.6.11 (University of Waikato, Hamilton, New Zealand). The leave-one-out cross-validation (LOOCV) method was applied to validate the model’s accuracy and robustness and to generalize the decision tree results.

The J48 algorithm inherently assesses variable importance during tree construction by using Information Gain to rank and split attributes. The importance of each attribute was subsequently interpreted topologically directly from the decision tree structure, where variables positioned closer to the root node represent parameters with the highest discriminative power.

## 3. Results

### 3.1. Clinical Characteristics of the Cohort

This study evaluated 96 women diagnosed with cervical cancer (cases) and 31 healthy volunteers with normal cytology and endovaginal ultrasound results. The clinical characteristics of the women are summarized in Table 1.

The median age was significantly higher in the cancer group (53.0 years) compared to the control group (45.4 years), *p* = 0.026. Patients were further stratified by age to examine extracellular vesicle profiles: 8 patients (8.3%) were under 35, 41 (42.7%) were between 35 and 55, and 47 (49.0%) were over 55 years. Family history of cancer was more prevalent in patients, with 43 (44.8%) reporting a family history compared to 7 (22.6%) in controls (*p* = 0.047).

In the cancer group, histological analysis identified squamous cell carcinoma in 76 patients (79.2%), adenocarcinoma in 19 (19.8%), and other rare histologies in 1 (1.0%). Clinical staging revealed advanced disease in most patients: 7 (7.3%) were stage I, 25 (26.0%) were stage II, 54 (56.3%) were stage III, and 10 (10.4%) were stage IV. Histopathological grading showed that 48 (50.0%) of patients had grade III tumors, 39 (40.6%) had grade II, and 9 (9.4%) had grade I.

Following eight months of chemotherapy and radiotherapy, 61 patients (63.5%) were non-recurrent, 16 (16.7%) experienced recurrence, and 19 (17.8%) were deceased. Parametrial involvement was detected in 86 patients (89.6%): 50 (52.1%) with bilateral involvement and 36 (37.5%) with unilateral involvement. Vaginal involvement was observed in 77 cases (80.2%). Regarding metastasis status before chemotherapy, nine patients (9.4%) had metastasis, 55 (57.3%) were metastasis-free, and metastasis status was unknown for 32 (33.3%).

This stratified data establishes a demographic and clinical profile foundation for interpreting EV profiles to age, histology, and disease progression.

### 3.2. Circulating EVs and Their Cell-Specific Profiles

This study examined the absolute counts of circulating EVs and their cell-specific profiles, including those derived from neutrophils, T lymphocytes, platelets, leukocytes, monocytes, erythrocytes, and endothelial cells, in two primary groups: women diagnosed with cervical cancer and a control group of healthy women with no cytological abnormalities.

To investigate associations between EV profiles and clinicopathological parameters, the cervical cancer group was subdivided. These subgroups were designed to assess survival outcomes (patients who responded to treatment, those who did not respond, and those who had passed away), clinical disease stage (early-stage patients in stages I and II versus advanced-stage patients in stages III and IV), and histopathological tumor grade (grades I, II, and III). This approach enabled assessment of how EV levels and cell-type profiles relate to treatment response, disease progression, and tumor aggressiveness.

### 3.3. Immunophenotypic Profiling of Circulating EVs in CC Patients: Evidence of Systemic Activation and Tumor Microenvironment Adaptation

**Figure 1:**
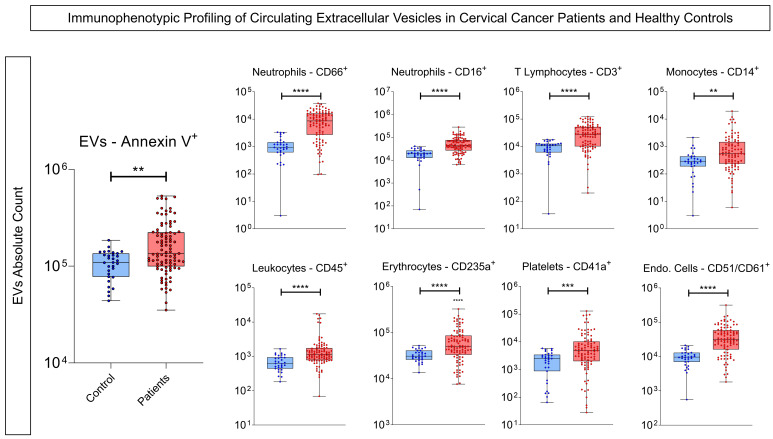
Immunophenotypic Profiling of Circulating Extracellular Vesicles in Cervical Cancer (CC) Patients and Healthy Controls. Absolute counts of circulating EVs were quantified and compared between cervical cancer patients and healthy control women. Circulating EV levels were measured in cervical cancer patients (*n* = 96) and compared with those of healthy women (*n* = 31). EV subpopulations were characterized using cell-specific markers: Annexin V^+^ (total EVs), CD66^+^ and CD16^+^ (neutrophil-derived EVs), CD3^+^ (T lymphocyte-derived EVs), CD14^+^ (monocyte-derived EVs), CD45^+^ (leukocyte-derived EVs), CD235a^+^ (erythrocyte-derived EVs), CD41a^+^ (platelet-derived EVs), and CD51/CD61^+^ (endothelial cell-derived EVs). Data are presented as box plots, indicating the median, interquartile range, and minimum/maximum values. Statistically significant differences between groups are denoted by asterisks (** *p* < 0.01, *** *p* < 0.001, **** *p* < 0.0001).

As shown in Figure 1, the immunophenotypic profiling of circulating EVs reveals a significant overall increase in EV release in cervical cancer patients compared to healthy controls, as indicated by a marked rise in Annexin V-positive EVs (*p* < 0.01). This elevation is accompanied by substantial increases in specific cell-type EV populations. Neutrophil-derived EVs, identified by CD66^+^ and CD16^+^ markers, showed the most pronounced elevation (*p* < 0.0001 for both markers), which may reflect heightened neutrophil activity or degranulation. Similarly, T lymphocytes (CD3^+^) and leukocyte-derived EVs (CD45^+^) were significantly elevated in cancer patients (*p* < 0.0001), suggesting an enhanced immune response potentially linked to tumor-associated antigens.

Monocyte-derived EVs (CD14^+^) and erythrocyte-derived EVs (CD235a^+^) were also elevated in cancer patients, with *p*-values < 0.01 and <0.001, respectively, suggesting innate immune activation and possible hemolysis or erythrocyte turnover in the observed increase in EV counts. Additionally, significant elevations in platelet-derived EVs (CD41a^+^) and endothelial cell-derived EVs (CD51/CD61^+^) (*p* < 0.0001 for both) highlight the involvement of vascular components, potentially reflecting endothelial activation or increased platelet turnover associated with cervical cancer.

Collectively, these data indicate a broad upregulation of circulating EVs from diverse cellular origins in cervical cancer patients. This systemic elevation may mirror immune activation, vascular stress responses, and other tumor microenvironment adaptations that could be critical in cancer progression.

### 3.4. Association of Extracellular Vesicle Profiles with Clinical Outcomes in Cervical Cancer Patients: Systemic Indicators of Tumor Presence

**Figure 2:**
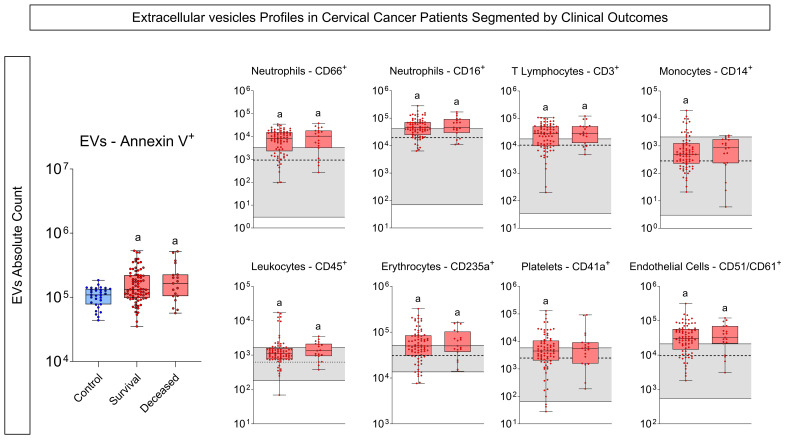
Extracellular vesicle Profiles in Cervical Cancer Patients Segmented by Clinical Outcomes. Circulating EV levels were measured in cervical cancer patients, stratified by outcome into survivors (*n* = 77) and non-survivors (*n* = 19), and compared with healthy women (*n* = 31). EV subpopulations were characterized using cell-specific markers: Annexin V^+^ (total EVs), CD66^+^ and CD16^+^ (neutrophils), CD3^+^ (T lymphocytes), CD14^+^ (monocytes), CD45^+^ (leukocytes), CD235a^+^ (erythrocytes), CD41a^+^ (platelets), and CD51/CD61^+^ (endothelial cells). Data are presented as box plots, showing median, interquartile range, and minimum and maximum values. Statistically significant differences between patient groups and controls are denoted by “a”.

Circulating EV profiles in cervical cancer patients were analyzed based on clinical outcomes, comparing survivors and deceased patients to a healthy control group. Total EV levels, indicated by Annexin V positivity, were significantly elevated in both the survivor and deceased groups relative to controls (*p* < 0.05 for both comparisons). However, no significant differences were observed between survivors and deceased patients, suggesting that the overall EV burden is similarly increased in both groups (Figure 2).

When analyzing cell-specific EV populations, significant increases were observed across EVs derived from neutrophils (CD66^+^ and CD16^+^), T lymphocytes (CD3^+^), leukocytes (CD45^+^), erythrocytes (CD235a^+^), and endothelial cells (CD51/CD61^+^), in both survivors and deceased patients compared to the control group (*p* < 0.05 for all markers). However, elevations in monocyte (CD14^+^) and platelet-derived (CD41a^+^) EVs were restricted to the survivor group compared with controls. These elevations were consistent across the two cancer subgroups, with no statistically significant differences detected between survivors and deceased patients for any of the evaluated EV populations.

These findings suggest that the systemic increase in circulating EVs observed in cervical cancer patients reflects a generalized biological response to the disease rather than being influenced by survival status. The consistent elevation of EVs derived from immune, vascular, and erythrocyte sources across both clinical outcomes highlights their potential role as biomarkers of cancer presence and systemic involvement, independent of prognosis.

### 3.5. Variations in Extracellular Vesicle Profiles Among Cervical Cancer Patients Stratified by Survival Outcomes: Potential Biomarkers for Treatment Response

**Figure 3:**
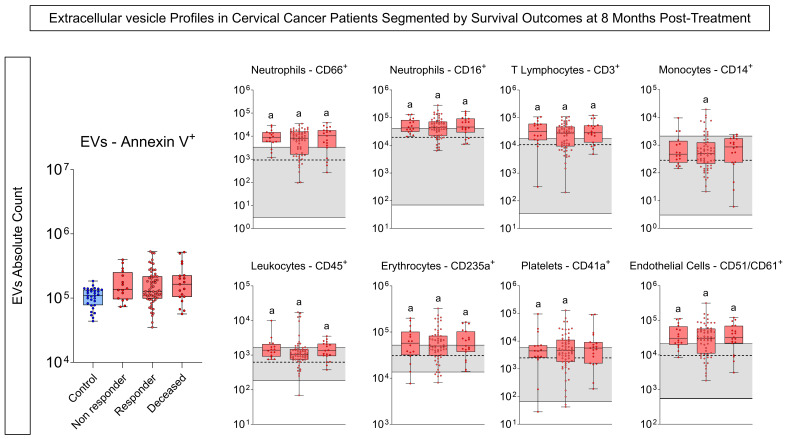
Extracellular vesicle Profiles in Cervical Cancer Patients Segmented by Survival Outcomes at 8 Months Post-Treatment. Circulating EV levels were assessed in cervical cancer patients, stratified by survival outcome at 8 months post-treatment into non-responders (*n* = 16), responders (*n* = 61), and deceased (*n* = 19), and compared with healthy women (*n* = 31). EV subpopulations were characterized using cell-specific markers: Annexin V^+^ (total EVs), CD66^+^ and CD16^+^ (neutrophils), CD3^+^ (T lymphocytes), CD14^+^ (monocytes), CD45^+^ (leukocytes), CD235a^+^ (erythrocytes), CD41a^+^ (platelets), and CD51/CD61^+^ (endothelial cells). Data are displayed as box plots, showing median, interquartile range, and minimum and maximum values. Statistically significant differences between cancer patient groups and controls are indicated by “a”.

When patients were stratified by 8-month survival outcomes (Figure 3), distinct circulating EV profiles emerged when comparing non-responders, responders, and deceased individuals to healthy controls. Total EV levels, indicated by Annexin V positivity, were significantly elevated across all cancer subgroups relative to controls. Non-responders (*p* = 0.0229), responders (*p* = 0.0092), and deceased patients (*p* = 0.0102) consistently exhibited statistically significant increases in total EVs, suggesting a broad systemic response that is independent of survival outcomes.

Cell-specific EV populations displayed significant changes across all cancer subgroups, particularly those derived from immune and vascular cells. EVs originating from neutrophils (CD66^+^ and CD16^+^) and T lymphocytes (CD3^+^) were significantly elevated across all cancer subgroups compared to controls (*p* < 0.0001). Monocyte-derived EVs (CD14^+^) showed a significant increase only in responders (*p* = 0.0073). In contrast, Leukocyte-derived EVs (CD45^+^) exhibited strong elevations in non-responders and responders (*p* < 0.0001 for both) and a more moderate rise in deceased patients (*p* = 0.0002). Platelet-derived EVs (CD41a^+^) were significantly higher in non-responders (*p* = 0.0178) and responders (*p* = 0.0008) but not in deceased patients, while endothelial-derived EVs (CD51/CD61^+^) displayed uniform and pronounced elevations across all clinical groups (*p* < 0.0001).

Erythrocyte-derived EVs (CD235a^+^) displayed a pattern of significant elevation across all cancer subgroups when compared to the control group. Non-responders (*p* = 0.0105) and deceased patients (*p* = 0.002) exhibited markedly higher levels relative to controls, indicating a systemic increase associated with disease progression. Responders, however, presented an even more pronounced elevation (*p* = 0.0002), suggesting that erythrocyte-derived EVs are not solely indicative of poor prognosis but may also reflect active biological processes independent of survival outcomes.

Collectively, these findings indicate that elevated EV levels from immune and vascular sources are characteristic of cancer-related systemic processes that persist across diverse survival outcomes. The consistent elevation of EVs from neutrophils, T lymphocytes, monocytes, platelets, and endothelial cells underscores the systemic nature of these responses, likely associated with tumor progression or recurrence. Notably, the distinct pattern observed in responders for erythrocyte-derived EVs highlights their potential as biomarkers for improved outcomes, supporting further investigation into their functional role in cancer progression and response to therapy.

### 3.6. Consistent Elevation of Circulating Extracellular Vesicles Across Early and Advanced Stages of Cervical Cancer: Potential Biomarkers for Tumor Progression

**Figure 4:**
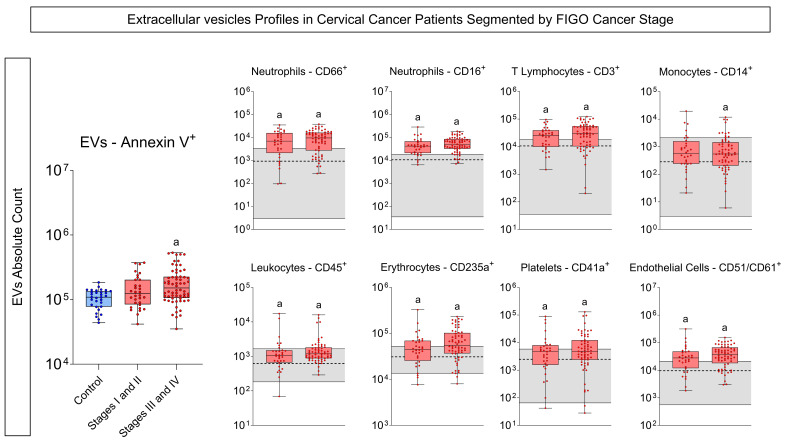
Extracellular vesicle Profiles in Cervical Cancer Patients Segmented by FIGO Cancer Stage. Circulating EV levels were assessed in cervical cancer patients, stratified by cancer stage into early-stage (stages I and II, *n* = 32) and advanced-stage (stages III and IV, *n* = 64), and compared with healthy women (*n* = 31). EV subpopulations were characterized using cell-specific markers: Annexin V^+^ (total EVs), CD66^+^ and CD16^+^ (neutrophils), CD3^+^ (T lymphocytes), CD14^+^ (monocytes), CD45^+^ (leukocytes), CD235a^+^ (erythrocytes), CD41a^+^ (platelets), and CD51/CD61^+^ (endothelial cells). Data are presented as box plots, showing median, interquartile range, and minimum and maximum values. Statistically significant differences between patient groups and controls are indicated by “a”.

As illustrated in Figure 4, circulating EV profiles were assessed according to clinical stage, comparing early-stage (I–II) and advanced-stage (III–IV) cervical cancer patients with healthy controls. Total EV levels, indicated by Annexin V positivity, were significantly elevated in advanced-stage patients (*p* < 0.05) compared to controls, while no significant difference was observed in early-stage patients relative to controls. This suggests that total EV levels may be more closely associated with advanced disease progression.

In the analysis of cell-specific EV populations, both early-stage and advanced-stage cervical cancer patients exhibited significant elevations in EVs derived from neutrophils (CD66^+^ and CD16^+^), T lymphocytes (CD3^+^), leukocytes (CD45^+^), erythrocytes (CD235a^+^), platelets (CD41a^+^), and endothelial cells (CD51/CD61^+^) compared to the control group (*p* < 0.05 for all markers). Importantly, monocyte-derived EVs (CD14^+^) showed a significant increase only in advanced-stage patients relative to controls (*p* < 0.05), with no difference observed in early-stage patients. No significant differences were observed between early- and advanced-stage groups for any of these EV populations, indicating that their elevation is a consistent feature across different stages of cervical cancer.

These findings highlight that elevated levels of EVs from diverse cellular origins are strongly associated with cervical cancer, regardless of disease stage. While the lack of a significant increase in total EVs in early-stage patients suggests a potential limitation of total EV counts as a diagnostic marker in early disease, the consistent elevation of cell-specific EVs across both stages underscores their potential as robust biomarkers for cervical cancer.

### 3.7. Extracellular Vesicle Profiles Across Histopathological Grades of Cervical Cancer: Implications for Tumor Diagnosis and Classification

**Figure 5:**
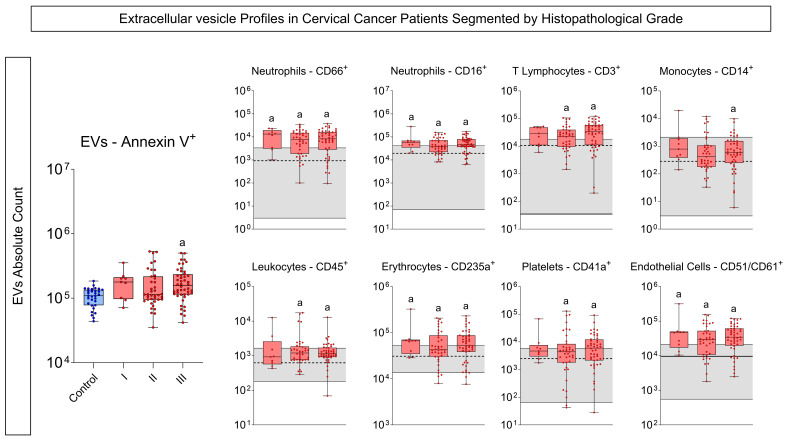
Extracellular vesicle Profiles in Cervical Cancer Patients Segmented by Histopathological Grade. Circulating extracellular vesicle numbers were assessed in cervical cancer patients stratified by histopathological tumor grade, grade I (*n* = 9), grade II (*n* = 39), and grade III (*n* = 48), and compared with healthy women (*n* = 31). EV subpopulations were characterized using cell-specific markers: Annexin V^+^ (total EVs), CD66^+^ and CD16^+^ (neutrophils), CD3^+^ (T lymphocytes), CD14^+^ (monocytes), CD45^+^ (leukocytes), CD235a^+^ (erythrocytes), CD41a^+^ (platelets), and CD51/CD61^+^ (endothelial cells). Data are displayed as box plots, showing median, interquartile range, and minimum and maximum values. Statistically significant differences between patient groups and controls are denoted by “a”.

The circulating EV profiles in cervical cancer patients were also analyzed according to histopathological tumor grade, comparing patients with grade I, II, and III tumors to a healthy control group. Total EVs, indicated by Annexin V positivity, were significantly elevated only in patients with grade III tumors (*p* < 0.05) compared to controls. In contrast, patients with grade II tumors did not show a statistically significant increase compared with controls. These results suggest that total EV levels are not consistently associated with histopathological grade, as illustrated in Figure 5.

In the analysis of cell-specific EV populations, most markers demonstrated significant elevations relative to controls, with specific variations depending on tumor grade. For EVs derived from neutrophils (CD66^+^ and CD16^+^), significant increases were observed in all tumor grades (I, II, and III) compared to the control group (*p* < 0.05 for all comparisons). T lymphocyte-derived EVs (CD3^+^) were significantly elevated only in grade II and grade III tumors, while monocyte-derived EVs (CD14^+^) were significantly increased only in grade III tumors. Similarly, Leukocyte-derived EVs (CD45^+^) and platelet-derived EVs (CD41a^+^) were significantly elevated in grade II and grade III tumors compared to controls, but not in grade I tumors. Erythrocyte-derived EVs (CD235a^+^) and endothelial-derived EVs (CD51/CD61^+^) were significantly increased in all tumor grades (*p* < 0.05 for all).

Importantly, no statistically significant differences were observed between tumor grades for any of the evaluated EV populations, indicating that their elevation is a consistent feature across different levels of tumor differentiation and aggressiveness. This consistency across grades suggests a general systemic response to the presence of cancer that does not vary significantly with tumor grade.

These findings demonstrate that increased levels of EVs from multiple cellular origins are a common feature among patients with cervical cancer. Although total EVs and some specific EV subsets (such as CD14^+^ and CD45^+^) exhibited grade-dependent variations in statistical significance, the overall pattern of elevation remained consistent across grades, reinforcing their potential as circulating markers indicative of tumor-associated cellular activation.

### 3.8. Signature Profile of Circulating EVs in CC Patients

**Figure 6:**
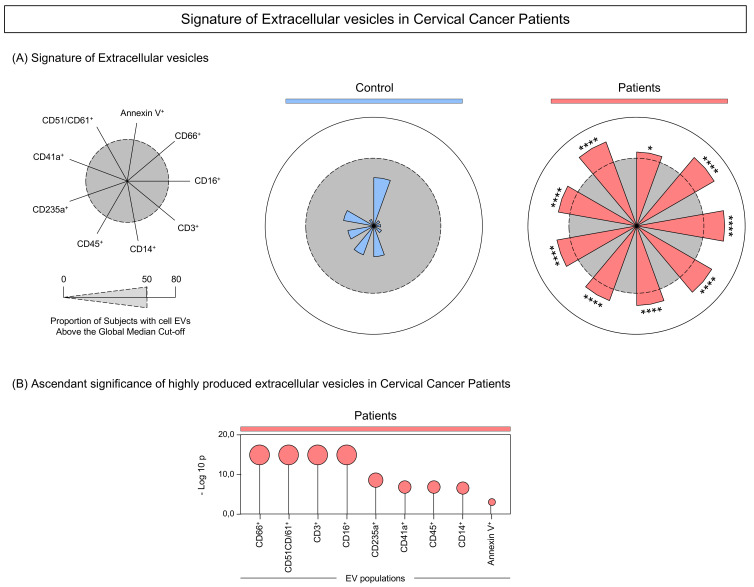
Signature and statistical significance of extracellular vesicles in colorectal cancer patients. (**A**) The overall signature of EV populations in the CC patients was assembled at baseline. Data, originally expressed as absolute numbers of EVs/mm^3^ of plasma, were converted into categorical data using the global median values, which were used as a cut-off point to classify the study population as being a low or high producer of the EVs evaluated. The overall signatures were assembled in radar charts using the 50th percentile as the threshold (central circle/gray zone) to identify EV populations with increased levels in a higher proportion of patients. The Fisher Exact Test was performed to evaluate the difference between the proportions of EVs in CC patients and Controls, and significant differences are highlighted by asterisks for *p* < 0.05 (*) or *p* < 0.0001 (****). (**B**) To assess the magnitude of the statistical significance of the biomarkers, the Delta value for the differences in the proportion of high-producing individuals of the EVs evaluated (CC patients in relation to the controls) was calculated. Then, Fisher’s Exact Test was performed, and the *p*-value was converted to −LOG10 to highlight the populations of EVs with greater statistical significance.

The populations of EVs were analyzed to identify the signature profile using the global median values for each phenotypic feature. CC patients had increased circulating EVs from all cell subsets when compared to controls (Figure 6).

### 3.9. CC Biomarkers Performance Index to Identify Subgroups of Patients with Cancer

**Figure 7:**
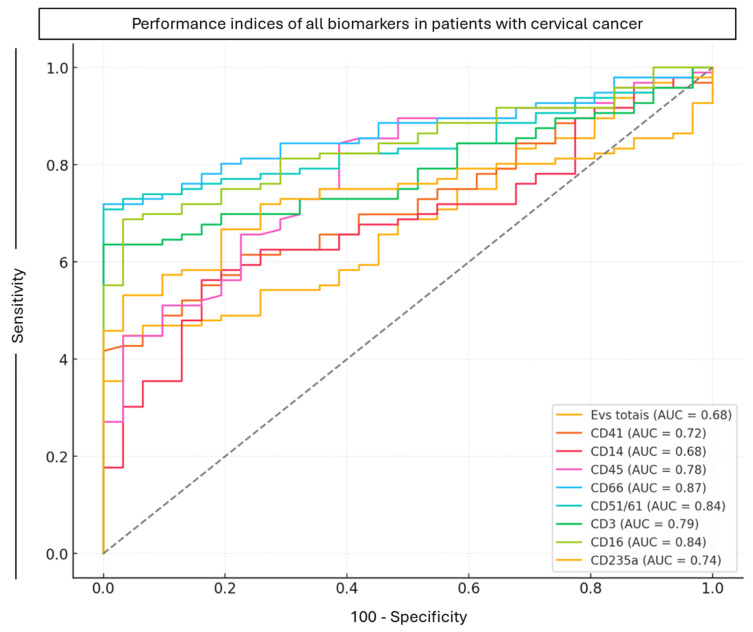
Receiver operating characteristic (ROC) curves illustrating the diagnostic performance of extracellular vesicle (EV)-associated biomarkers in patients with cervical cancer. The area under the curve (AUC) values are provided for each biomarker, indicating their respective discriminatory capacities. CD66^+^ exhibited the highest AUC (0.87), followed by CD51/CD61^+^ and CD16^+^ (both with AUC = 0.84), CD3^+^ (AUC = 0.79), CD45^+^ (AUC = 0.78), CD41^+^ (AUC = 0.72), and CD235a^+^ (AUC = 0.74). The total EV population demonstrated the lowest diagnostic accuracy (AUC = 0.68). The dashed diagonal line represents the reference (random classifier) line.

A comparative analysis was performed to benchmark the diagnostic performance of all EV subpopulations. To evaluate the diagnostic potential of EV populations, receiver operating characteristic (ROC) curves were generated for all markers, and their respective area under the curve (AUC) values were calculated (Figure 7). These initial analyses provided an overview of biomarker performance, allowing the identification of those with the highest discriminatory capacity in distinguishing patients with cervical cancer. This head-to-head assessment allowed the identification of the most discriminative markers, which were then selected for further analysis.

Based on these findings, only the biomarkers with the highest AUC values were selected for further analysis: CD66^+^ (AUC = 0.868), CD16^+^ (AUC = 0.840), CD51/CD61^+^ (AUC = 0.840), and CD3^+^ (AUC = 0.786).

**Figure 8:**
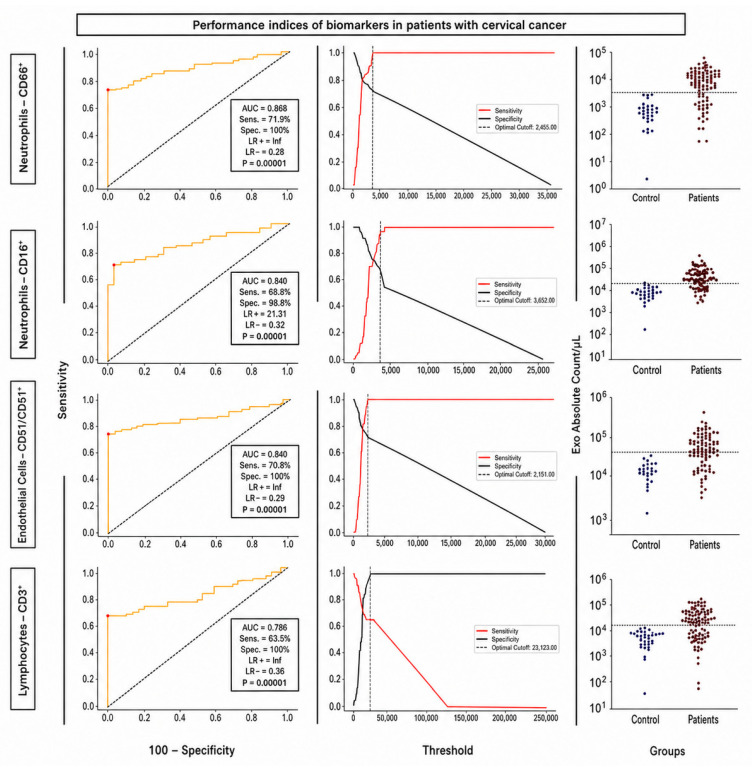
Comparative diagnostic performance of EV biomarkers in cervical cancer. The figure presents the diagnostic accuracy of extracellular vesicles derived from CD66^+^ neutrophils, CD16^+^ neutrophils, CD51/CD61^+^ endothelial cells, and CD3^+^ lymphocytes in patients with cervical cancer. The left panel displays receiver operating characteristic (ROC) curves comparing sensitivity, specificity, and area under the curve (AUC) values across markers, including optimal cut-off thresholds, likelihood ratios [LR(+), LR(–)], and corresponding *p*-values. The middle panel illustrates two-dimensional threshold-gradient ROC (TG-ROC) plots, showing the relationship between sensitivity and specificity across varying cut-off points. The right panel depicts the distribution of individual EV marker levels stratified by diagnostic status and optimal cut-off, facilitating direct visualization of each marker’s discriminative capacity.

To further explore the clinical applicability of these markers, additional analyses were performed, incorporating sensitivity, specificity, likelihood ratio (LR+), and optimal cut-off values determined using Youden’s index. Figure 8 provides a detailed breakdown of the selected biomarkers, including their respective performance metrics and statistical significance.

CD66^+^ exhibited the highest diagnostic performance, with an area under the curve (AUC) of 0.868, a sensitivity of 71.9%, and a specificity of 100%, corresponding to an optimal cut-off value of 3365. The likelihood ratio for a positive test (LR^+^) tended toward infinity, and the statistical significance was robust (*p* < 0.00001).

CD16^+^ and CD51/CD61^+^ demonstrated comparable discriminatory capacity, presenting an AUC of 0.840. CD16^+^ showed a sensitivity of 68.8% and a specificity of 96.8%, with a cut-off value of 36,012 and an LR^+^ of 0.32. In contrast, CD51/CD61^+^ had a sensitivity of 70.8%, a specificity of 100%, a cut-off value of 21,351, and an LR^+^ tending toward infinity. Both markers achieved high statistical significance (*p* < 0.00001).

Finally, CD3^+^ exhibited an AUC of 0.786, a sensitivity of 63.5%, and a specificity of 100%, with an optimal cut-off value of 22,112. The LR^+^ tended toward infinity, and the association remained statistically significant (*p* < 0.00001).

A final dispersion analysis was conducted to validate the clinical relevance of these markers. It demonstrated that the optimized cut-off values effectively separate patient groups based on biomarker expression. These findings suggest that these markers may be potential candidates for immune profiling in cervical cancer. Taken together, the comparative analysis highlights the superior diagnostic performance of CD66^+^, followed by CD16^+^, CD51/CD61^+^, and CD3^+^ EVs, underscoring their translational potential as biomarkers of cervical cancer.

### 3.10. Development of a Decision Tree Algorithm for the Classification of Cervical Cancer Patients

To enhance patient stratification, three distinct decision tree algorithms were developed, each designed to address a specific clinical question: (i) classification of CC patients versus healthy controls, (ii) prediction of clinical outcomes (survival vs. mortality), and (iii) assessment of treatment response.

### 3.11. Classification of Cervical Cancer Patients and Healthy Controls

**Figure 9:**
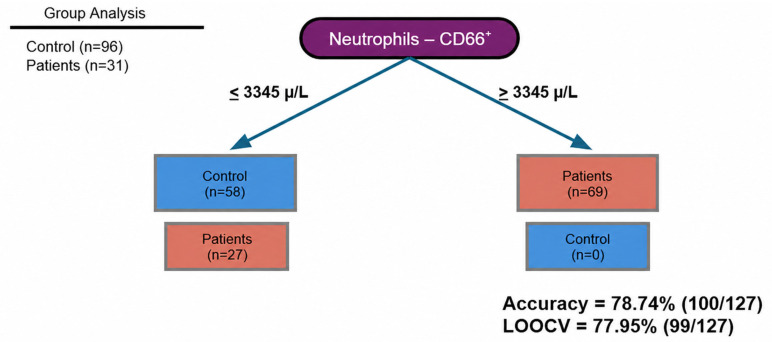
Decision tree algorithm to classify cervical cancer patients and healthy controls. Decision tree model based on neutrophil-derived extracellular vesicles (CD66^+^) for the classification of cervical cancer patients and healthy controls. A decision tree algorithm (J48) was developed using flow cytometry data from circulating neutrophil-derived extracellular vesicles (CD66^+^) to discriminate cervical cancer patients (*n* = 31) from healthy controls (*n* = 96). The model identified CD66^+^ EVs as the most informative attribute, with an optimal cut-off threshold of 3345 µL^−1^, determined using information gain criteria. Individuals presenting CD66^+^ EV concentrations ≥ 3345 µL^−1^ were classified as Cervical Cancer patients (*n* = 69; no misclassified controls), while those with values ≤ 3345 µL^−1^ were primarily assigned to the Healthy Control group (*n* = 58), with 27 cases classified as Cervical Cancer patients. The overall model achieved an accuracy of 78.74% (100/127) and a LOOCV accuracy of 77.95% (99/127), confirming its internal robustness and reproducibility. This analysis highlights the high discriminatory capacity of CD66^+^ neutrophil-derived EVs as a single biomarker for cervical cancer detection.

A decision tree model was constructed to differentiate CC patients from healthy individuals. This classifier exhibited a high overall accuracy of 78.74% (100/127) and maintained robust predictive performance with a leave-one-out cross-validation accuracy of 77.95% (99/127) (Figure 9). The model leveraged a single key biomarker, CD66^+^ neutrophils, to achieve optimal discrimination.

## 4. Discussion

Cervical cancer remains a major global health concern, particularly in low- and middle-income countries, where deficiencies in preventive infrastructure and delays in diagnosis and treatment contribute to persistently high morbidity and mortality rates [1]. Despite the implementation of HPV vaccination programs and cytological screening in high-income regions, a substantial proportion of cases in vulnerable populations continue to be diagnosed at advanced stages, limiting treatment efficacy and compromising long-term survival [3]. This enduring disparity underscores the urgent need for innovative diagnostic strategies that are both biologically informative and logistically feasible in resource-limited settings.

Building on their emerging role in tumor biology, EVs have garnered substantial interest as clinically relevant biomarkers in oncology. These membrane-bound particles, actively secreted by virtually all cell types under physiological and pathological conditions, circulate stably in body fluids and can be isolated through minimally invasive procedures [5]. Their lipid bilayer encapsulates a diverse array of biomolecules, including proteins, mRNAs, and microRNAs, that reflect the molecular landscape of their cells of origin [17]. Beyond serving as passive indicators, EVs actively participate in cancer progression by transporting oncogenic signals that modulate angiogenesis, immune evasion, epithelial–mesenchymal transition, and metastasis [18]. This dual function, as both effectors and reporters of tumor biology, positions EVs as powerful tools for integrated diagnostic, prognostic, and therapeutic monitoring in cancers such as cervical carcinoma, where non-invasive and biologically relevant biomarkers are urgently needed [13].

Recent evidence indicates that global EV expansion is not unique to cervical cancer but rather represents a common axis of tumor-driven systemic activation across malignant entities. In acute myeloid leukemia (AML), Szczepanski et al. (2011) recorded a surge of blast-derived microvesicles bearing transforming growth factor-β1 that suppressed natural-killer cytotoxicity [19]; Hong et al. (2014) later reported post-induction declines and consolidation-phase rebounds tightly linked to residual disease [20]. In pediatric B-cell acute lymphoblastic leukemia (B-ALL), Magalhães-Gama et al. (2024) observed a five-fold increase in CD10^+^CD19^+^ EVs at diagnosis with a stepwise fall during therapy [14]. Extending this pattern to solid tumors, Moreira et al. (2025) found marked elevations of neutrophil- (CD66^+^/CD16^+^) and endothelial-derived (CD51/CD61^+^) microvesicles in breast carcinoma, which contracted after treatment yet rebounded with residual disease [11]. Collectively, these studies confirm the ubiquity of quantitative EV surges; however, the phenotypic signature remains disease-specific, reinforcing lineage-oriented profiling for inter-tumor discrimination.

Although Annexin V^+^ EVs were significantly elevated in our cervical cancer cohort, their standalone diagnostic value was modest (AUC = 0.68) and non-significant in early disease. Similar limitations were reported by Morasso et al. (2022), who noted poor specificity for bulk CD9^+^/CD63^+^ vesicles in early breast cancer [21], and by Uthamacumaran et al. (2022), whose fluorescence-correlation approach achieved 90% accuracy but relied on heterogeneous bulk EV preparations [22].

By contrast, our multiparametric flow-cytometry strategy revealed a robust and reproducible elevation of EVs derived from neutrophils (CD66^+^, CD16^+^), T lymphocytes (CD3^+^), monocytes (CD14^+^), leukocytes (CD45+), platelets (CD41a^+^), erythrocytes (CD235a^+^), and endothelial cells (CD51/CD61^+^), with most *p*-values below 0.0001. Among them, CD66^+^ neutrophil EVs yielded the best diagnostic performance (AUC = 0.868; 100% specificity; 71.9% sensitivity). A single-marker decision tree surpassed 78% accuracy and 77.9% cross-validation, rivaling the multi-protein ACE panel of Hinestrosa et al. (2022) (AUC = 0.95) [23] while demanding far less analytical complexity.

Beyond their superior metrics, neutrophil- and endothelial-derived vesicles displayed remarkable uniformity across the entire clinical spectrum we examined. They were already prominent in early-stage and low-grade disease and remained comparably abundant in advanced tumors, irrespective of short-term therapeutic response or survival status. This stability indicates that cervical-cancer-driven granulocytic and endothelial activation is established at tumor onset and imprints a durable vesicular signature that bulk Annexin V measurements fail to capture, underscoring the unique informational value of lineage-resolved profiling.

Although this study was not primarily designed to assess long-term prognostic endpoints, exploratory analyses indicated that the elevated levels of neutrophil- and endothelial-derived EVs were consistently observed across patients regardless of short-term therapeutic response or follow-up status, suggesting a stable vesicular signature associated with tumor burden and systemic engagement. These findings, when interpreted alongside prior longitudinal evidence from hematologic and breast malignancies, reinforce the potential of lineage-defined EVs as dynamic biomarkers with both diagnostic and prognostic implications.

The translational appeal of EVs extends beyond diagnosis, as longitudinal studies consistently demonstrate that vesicular dynamics mirror disease evolution. In AML, Szczepanski et al. (2011) and Hong et al. (2014) observed that exosome-bound transforming growth factor β1 declines after induction chemotherapy and resurges during consolidation, signaling remission and subsequent minimal residual disease [19,20]. In padiatric B-ALL, Magalhães-Gama et al. (2024) reported a progressive normalization of EV networks that paralleled the reduction in leukemic burden [14]. A comparable pattern has been documented in breast carcinoma, where Moreira et al. (2025) showed that neutrophil- and endothelial-derived microvesicles contract post-therapy and transiently rebound in the presence of residual disease [11]. Prospective serial quantification of CD66^+^ EVs could therefore validate their utility as minimally invasive sentinels for prognosis, risk stratification, and treatment monitoring.

Taken together, these insights confirm that while EV quantity rises across malignancies, cell-of-origin signatures provide the discriminatory power. The neutrophil-centered vesicular pattern identified here, coupled with its strong diagnostic metrics, advances the field of EV-based biomarkers for cervical cancer.

In summary, directed immunophenotypic profiling outperforms bulk EV measurement, offering a sensitive, specific, and scalable approach for disease detection and longitudinal surveillance. The translational leverage of these findings lies in the identification of lineage-defined EVs as actionable biomarkers for cervical cancer, a feature that expands the toolbox for risk stratification and personalized management. Routine incorporation of EV profiling could accelerate detection of aggressive phenotypes and refine therapeutic choices, especially in advanced-stage tumors or in patients who exhibit suboptimal responses to conventional regimens. Unlike static protein markers, EVs provide a dynamic window into tumor biology, immune modulation, and systemic activity. Their minimally invasive accessibility renders them particularly valuable where repeated tissue sampling is unfeasible, thereby reinforcing their suitability for low-resource settings. Collectively, the present data establish a foundation for EV-centered platforms that complement existing screening tools and support real-time clinical decision-making in cervical-cancer care.

Despite these promising results, certain limitations must be acknowledged. The single-center design and modest cohort size limit the external generalizability of our findings. Validation in larger, multicentric, and demographically diverse populations is essential to confirm the robustness of the proposed EV-based biomarkers. Furthermore, the cross-sectional nature of this study precludes inferences about temporal dynamics or causal relationships between EV profiles and clinical outcomes. Longitudinal studies are warranted to assess the prognostic utility of these vesicular signatures throughout treatment and disease evolution. Another critical challenge is the methodological heterogeneity in EV isolation and characterization across the literature. The lack of standardized protocols currently hampers reproducibility and translation into clinical workflows. Establishing consensus guidelines akin to those used for circulating tumor DNA or protein-based assays will be a necessary step toward clinical implementation.

### 4.1. Conclusions

Our findings support the concept that circulating extracellular vesicles, particularly those with defined cellular lineage markers, serve as reliable and informative indicators of cervical cancer presence and systemic engagement. More than inert byproducts, EVs represent a complex communication network that echoes the biological narrative of the tumor and its interaction with the host. By encapsulating oncogenic signals, immune modulators, and microenvironmental cues, they act not only as biomarkers but also as potential participants in disease progression.

Realizing the full translational potential of EVs will depend on overcoming current technical barriers and advancing our understanding of their biogenesis, heterogeneity, and functional roles in cancer. The development of scalable, standardized, and cost-effective EV-based technologies could redefine how we screen for, monitor, and manage cancer. As part of a broader shift toward precision medicine, EVs may ultimately help us deliver more accurate, timely, and personalized care, aligning diagnostics with the biological realities of each patient’s disease.

### 4.2. Limitations

While the present analysis demonstrated consistent elevations of lineage-specific EV populations across clinical stages, we acknowledge that more granular assessments incorporating tumor size (T) and lymph node status (N) were not performed due to limited subgroup representation. Future studies employing larger and more stratified cohorts will be essential to determine whether these clinical variables independently influence circulating EV levels. Nevertheless, the reproducible pattern of elevation across multiple immune and vascular EV subsets observed here argues against random variability and supports a genuine biological phenomenon linked to systemic tumor activity.

## Figures and Tables

**Table 1:** Clinical and pathological characteristics of the cohort.

Characteristics	Control Group	CC Patients
	*n* (%)	*n* (%)
Total	31 (100)	96 (100)
Age in years, median (IQR)	45 (25–70)	53 (29–80)
Age range		
<35	6 (19)	8 (8)
35–55	12 (39)	41 (43)
>55	13 (42)	47 (49)
Family history of cancers		
Absent	24 (77)	53 (55)
Present	7 (23)	43 (45)
Histological diagnosis		
Squamous cell carcinoma	N/A	76 (79)
Adenocarcinoma	N/A	19 (20)
Other cancers	N/A	1 (1)
Clinical staging based on the TNM system		
FIGO I	N/A	7 (7)
FIGO II	N/A	25 (26)
FIGO III	N/A	54 (56)
FIGO IV	N/A	10 (11)
Histological grade		
I	N/A	9 (9)
II	N/A	39 (41)
III	N/A	48 (50)
N/A	N/A	0 (0)
Status after 8 months of chemotherapy and radiotherapy		
No recurrence	N/A	61 (64)
Recurrence	N/A	16 (17)
Deceased	N/A	19 (18)
Parametrial involvement		
Absent	N/A	10 (10)
Unilateral	N/A	36 (38)
Bilateral	N/A	50 (52)
Vaginal Involvement		
Absent	N/A	19 (20)
Present	N/A	77 (80)
Metastasis before chemotherapy treatment		
Absent	N/A	55 (57)
Present	N/A	9 (10)
N/A	N/A	32 (33)

## Data Availability

The data presented in this study are available upon request to the corresponding author due to participant confidentiality.

## Supplementary Materials

The following supporting information can be downloaded at https://www.mdpi.com/article/10.3390/biomedicines14071492/s1. Figure S1. Representative strategy for the phenotypic characterization of extracellular vesicles (EV) by flow cytometry according to size range. (a) The histogram shows two peaks of different sizes of beads, representing 100 nm and 900 nm, respectively. (b) Histogram representing an sample (green) overlayed on different sizes of beads (100 nm and 900nm, respectively). (c) Two-dimensional plot of Annexin V-FITC vs Violet SSC-H with EV point distribution of size between 100 and 900 nm in the control tube (Annexin V buffer + Sample) allows positioning of the positivity region for Annexin V-FITC with a lower threshold at 4.5%. This selected region is applied to all immunophenotyping tubes. (d) One-way fluorescence intensity histograms are used for the quantification of phenotype-specific EV in percentage using the control tube (gray curves) to position the positivity threshold marker (color curves). The results are expressed in EV number/mm^3^, considering the aspirated volume in 2 min of acquisition. (e) Superimposed histograms of the fluorescences used, from negative and positive samples, which demonstrate the positioning of the gate after the negative (gray) peak, with the part of the colored peak that enters the gate being the positivity for that fluorescence; Figure S2. Representative Analysis for the Phenotypic Characterization of EVs in the Healthy Controls; Figure S3. Representative Analysis for the Phenotypic Characterization of EVs in the Patient Cohort; Table S1. Pairwise comparisons of extracellular vesicle markers between control vs. cervical cancer patients. Table S2. Global Kruskal–Wallis test for clinical-outcome comparisons. Table S3. Pairwise Dunn’s post hoc comparisons of circulating extracellular vesicle marker abundances across control, survivor, and deceased cohorts. Table S4. Global Kruskal–Wallis test for treatment response comparisons. Table S5. Pairwise Dunn’s post hoc comparisons of circulating extracellular vesicle marker abundances across control, non-responder, responder, and deceased cohorts. Table S6. Global Kruskal–Wallis test for clinical-stage comparisons. Table S7. Pairwise Dunn’s post hoc comparisons of circulating extracellular vesicle marker abundances across control, stage I–II and stage III–IV cohorts. Table S8. Global Kruskal–Wallis test for histopathological-grade comparisons. Table S9. Pairwise Dunn’s post hoc comparisons of circulating extracellular vesicle marker abundances across control and histopathological grade subgroups. All tests were two-tailed; significance thresholds were Bonferroni-adjusted for multiple comparisons (α = 0.05). Significance levels: ns > 0.05; * ≤ 0.05; ** ≤ 0.01; *** ≤ 0.001; **** ≤ 0.0001.
