# Supplementary material for: Elevated Circulating Extracellular Vesicles as Prognostic Biomarkers in Cervical Cancer Progression
Source: Biomedicines. 2026 Jun 30;14(7):1492. doi: 10.3390/biomedicines14071492 (PMC13404281; doi:10.3390/biomedicines14071492)
Supplement: Supplementary file 1 [file biomedicines-14-01492-s001.zip › biomedicines-3674133-Suplementary_Tables.pdf]

Supplementary Table S1. Pairwise comparisons of extracellular vesicle markers between control vs. cervical cancer patients

EV Marker	Control vs. Patients
**Total EVs (Annexin V^+)**	$p = 0.0021$
**$CD66^+$ EVs**	$p < 0.0001$
**$CD16^+$ EVs**	$p < 0.0001$
**$CD3^+$ EVs**	$p < 0.0001$
**$CD14^+$ EVs**	$p = 0.0022$
**$CD45^+$ EVs**	$p < 0.0001$
**$CD235a^+$ EVs**	$p < 0.0001$
**$CD41a^+$ EVs**	$p = 0.0002$
**$CD51/61^+$ EVs**	$p < 0.0001$

Supplementary Table S2 – Global Kruskal–Wallis test for clinical-outcome comparisons

Variable	H (χ^2)	p	Significance
**EVs_Total_Annexin_V**	9.532	0.0085	**
**CD66**	37.867	< 0.0001	****
**CD16**	32.465	< 0.0001	****
**CD3**	23.472	< 0.0001	****
**CD14**	9.435	0.0089	**
**CD45**	22.407	< 0.0001	****
**CD235a**	15.718	0.0004	***
**CD41a**	13.670	0.0011	**
**CD51_CD61**	32.303	< 0.0001	****

H, Kruskal–Wallis χ^2 statistic; p, two-tailed p value; significance levels: ns > 0.05; * ≤ 0.05; ** ≤ 0.01; * ≤ 0.001; **** ≤ 0.0001.**

Supplementary Table S3. Pairwise Dunn's post hoc comparisons of circulating extracellular vesicle marker abundances across control, survivor, and deceased cohorts.

Variable	Comparison	Mean rank diff	p (Bonf. adj.)	Significance
**EVs_Total_Annexin_V**	Control vs. Survival	-22.1500	0.0140	*
**EVs_Total_Annexin_V**	Control vs. Deceased	-27.1200	0.0344	*
**EVs_Total_Annexin_V**	Survival vs. Deceased	-4.9690	1.0000	ns
**CD66**	Control vs. Survival	-46.1500	< 0.0001	****
**CD66**	Control vs. Deceased	-49.0600	< 0.0001	****
**CD66**	Survival vs. Deceased	-2.9060	1.0000	ns
**CD16**	Control vs. Survival	-53.4200	< 0.0001	****
**CD16**	Control vs. Deceased	-45.8600	< 0.0001	****
**CD16**	Survival vs. Deceased	7.5700	1.0000	ns
**CD3**	Control vs. Survival	-43.4500	< 0.0001	****
**CD3**	Control vs. Deceased	-43.5100	< 0.0001	****
**CD3**	Survival vs. Deceased	-0.0553	1.0000	ns
**CD14**	Control vs. Survival	-27.8500	0.0134	*
**CD14**	Control vs. Deceased	-21.4000	0.1237	ns
**CD14**	Survival vs. Deceased	6.4510	1.0000	ns
**CD45**	Control vs. Survival	-42.6300	< 0.0001	****
**CD45**	Control vs. Deceased	-47.9300	< 0.0001	****
**CD45**	Survival vs. Deceased	-5.3010	1.0000	ns
**CD235a**	Control vs. Survival	-35.8300	0.0008	***
**CD235a**	Control vs. Deceased	-29.0700	0.0167	*
**CD235a**	Survival vs. Deceased	6.7640	1.0000	ns
**CD41a**	Control vs. Survival	-33.1800	0.0021	**
**CD41a**	Control vs. Deceased	-24.9800	0.0516	ns
**CD41a**	Survival vs. Deceased	8.1950	0.9725	ns
**CD51_CD61**	Control vs. Survival	-53.6900	< 0.0001	****
**CD51_CD61**	Control vs. Deceased	-43.4500	0.0001	***
**CD51_CD61**	Survival vs. Deceased	10.2400	0.6535	ns

Pairwise Dunn's test with tie and continuity corrections (0.5), Bonferroni-adjusted for three planned comparisons per marker ($\alpha_{adj} = 0.05/3 = 0.0167$); p (Bonf. adj.), Bonferroni-corrected two-tailed p; significance levels: ns > 0.05; * ≤ 0.05; ** ≤ 0.01; * ≤ 0.001; **** ≤ 0.0001.**

Supplementary Table S4 – Global Kruskal–Wallis test for treatment response comparisons

Variable	H (χ^2)	p	Significance
**EVs_Total_Annexin_V**	9.674	0.0215	*
**CD66**	38.434	< 0.0001	****
**CD16**	32.769	< 0.0001	****
**CD3**	25.104	< 0.0001	****
**CD14**	9.505	0.0233	*
**CD45**	25.024	< 0.0001	****
**CD235a**	15.722	0.0013	***
**CD41a**	13.686	0.0034	**
**CD51_CD61**	32.687	< 0.0001	****

H, Kruskal–Wallis χ^2 statistic; p, two-tailed p value; significance levels: ns > 0.05; * ≤ 0.05; ** ≤ 0.01; * ≤ 0.001; **** ≤ 0.0001.**

Supplementary Table S5. Pairwise Dunn's post hoc comparisons of circulating extracellular vesicle marker abundances across control, non-responder, responder, and deceased cohorts

Variable	Comparison	Mean rank diff	p (Bonf. adj.)	Significance
**EVs_Total_Annexin_V**	Control vs. Non_Responder	-25.23	0.1556	n.s.
**EVs_Total_Annexin_V**	Control vs. Responder	-21.34	0.0515	n.s.
**EVs_Total_Annexin_V**	Control vs. Deceased	-27.12	0.0687	n.s.
**EVs_Total_Annexin_V**	Non_Responder vs. Responder	3.898	1	n.s.
**EVs_Total_Annexin_V**	Non_Responder vs. Deceased	-1.882	1	n.s.
**EVs_Total_Annexin_V**	Responder vs. Deceased	-5.779	1	n.s.
**CD66**	Control vs. Non_Responder	-52.32	<0.0001	***
**CD66**	Control vs. Responder	-44.54	<0.0001	***
**CD66**	Control vs. Deceased	-49.06	<0.0001	***
**CD66**	Non_Responder vs. Responder	7.787	1	n.s.
**CD66**	Non_Responder vs. Deceased	3.263	1	n.s.
**CD66**	Responder vs. Deceased	-4.524	1	n.s.
**CD16**	Control vs. Non_Responder	-47.05	0.0002	***
**CD16**	Control vs. Responder	-41.35	<0.0001	***
**CD16**	Control vs. Deceased	-46.05	<0.0001	***
**CD16**	Non_Responder vs. Responder	5.703	1	n.s.
**CD16**	Non_Responder vs. Deceased	1.007	1	n.s.
**CD16**	Responder vs. Deceased	-4.696	1	n.s.
**CD3**	Control vs. Non_Responder	-45.26	0.0004	***
**CD3**	Control vs. Responder	-32.06	0.0005	***
**CD3**	Control vs. Deceased	-42.45	0.0005	***
**CD3**	Non_Responder vs. Responder	13.21	1	n.s.
**CD3**	Non_Responder vs. Deceased	2.813	1	n.s.
**CD3**	Responder vs. Deceased	-10.39	1	n.s.
**CD14**	Control vs. Non_Responder	-24.29	0.1921	n.s.
**CD14**	Control vs. Responder	-21.54	0.0478	*
**CD14**	Control vs. Deceased	-26.81	0.0744	n.s.
**CD14**	Non_Responder vs. Responder	2.752	1	n.s.
**CD14**	Non_Responder vs. Deceased	-2.52	1	n.s.
**CD14**	Responder vs. Deceased	-5.272	1	n.s.
**CD45**	Control vs. Non_Responder	-47.34	0.0002	***
**CD45**	Control vs. Responder	-30.63	0.001	**
**CD45**	Control vs. Deceased	-41.4	0.0007	***
**CD45**	Non_Responder vs. Responder	16.71	0.6365	n.s.
**CD45**	Non_Responder vs. Deceased	5.942	1	n.s.
**CD45**	Responder vs. Deceased	-10.76	1	n.s.
**CD235a**	Control vs. Non_Responder	-29.9	0.0499	*
**CD235a**	Control vs. Responder	-29.32	0.0018	**
**CD235a**	Control vs. Deceased	-32.5	0.0146	*
**CD235a**	Non_Responder vs. Responder	0.5809	1	n.s.
**CD235a**	Non_Responder vs. Deceased	-2.602	1	n.s.
**CD235a**	Responder vs. Deceased	-3.183	1	n.s.
**CD41a**	Control vs. Non_Responder	-26.02	0.1299	n.s.
**CD41a**	Control vs. Responder	-27.33	0.0046	**
**CD41a**	Control vs. Deceased	-31.24	0.0215	*

**CD41a**	Non_Responder vs. Responder	-1.317	1	n.s.
**CD41a**	Non_Responder vs. Deceased	-5.22	1	n.s.
**CD41a**	Responder vs. Deceased	-3.904	1	n.s.
**CD51_CD61**	Control vs. Non_Responder	-47.9	<0.0001	***
**CD51_CD61**	Control vs. Responder	-41.48	<0.0001	***
**CD51_CD61**	Control vs. Deceased	-44.7	0.0002	***
**CD51_CD61**	Non_Responder vs. Responder	6.414	1	n.s.
**CD51_CD61**	Non_Responder vs. Deceased	3.197	1	n.s.
**CD51_CD61**	Responder vs. Deceased	-3.217	1	n.s.

Pairwise Dunn's test with tie and continuity corrections (0.5); p (Bonf. adj.), Bonferroni-corrected two-tailed p (α_adj = 0.05/3 = 0.0167); significance levels: ns > 0.05; * ≤ 0.05; ** ≤ 0.01; * ≤ 0.001; **** ≤ 0.0001.**

Supplementary Table S6 – Global Kruskal–Wallis test for clinical-stage comparisons

Variable	H (χ^2)	p	Significance
**EVs_Total_Annexin_V**	11.405	0.0033	**
**CD66**	38.241	< 0.0001	****
**CD16**	33.352	< 0.0001	****
**CD3**	23.816	< 0.0001	****
**CD14**	9.533	0.0085	**
**CD45**	24.068	< 0.0001	****
**CD235a**	17.593	0.0002	***
**CD41a**	13.875	0.0010	***
**CD51_CD61**	33.471	< 0.0001	****

H, Kruskal–Wallis χ^2 statistic; p, two-tailed p value; significance levels: ns > 0.05; * ≤ 0.05; ** ≤ 0.01; * ≤ 0.001; **** ≤ 0.0001.**

Supplementary Table S7. Pairwise Dunn's post hoc comparisons of circulating extracellular vesicle marker abundances across control, stage I-II and stage III-IV cohorts.

Variable	Pairwise comparison	Mean rank diff	p (Bonf. adj.)	Significance
**EVs_Total_Annexin_V**	Control vs Stage I–II	–16.1951	0.2424	ns
	Control vs Stage III–IV	–27.1091	0.0023	**
	Stage I–II vs Stage III–IV	–10.9141	0.5124	ns
**CD66**	Control vs Stage I–II	–43.0882	< 0.0001	****
	Control vs Stage III–IV	–48.5491	< 0.0001	****
	Stage I–II vs Stage III–IV	–5.4609	1.0000	ns
**CD16**	Control vs Stage I–II	–37.8493	0.0001	***
	Control vs Stage III–IV	–45.9196	< 0.0001	****
	Stage I–II vs Stage III–IV	–8.0703	0.9335	ns
**CD3**	Control vs Stage I–II	–30.9985	0.0025	**
	Control vs Stage III–IV	–38.9751	< 0.0001	****
	Stage I–II vs Stage III–IV	–7.9766	0.9505	ns
**CD14**	Control vs Stage I–II	–24.8180	0.0224	ns
	Control vs Stage III–IV	–22.6696	0.0146	*
	Stage I–II vs Stage III–IV	2.1484	1.0000	ns
**CD45**	Control vs Stage I–II	–27.6200	0.0086	**
	Control vs Stage III–IV	–39.4481	< 0.0001	****
	Stage I–II vs Stage III–IV	–11.8281	0.4116	ns
**CD235a**	Control vs Stage I–II	–22.6951	0.0432	*
	Control vs Stage III–IV	–33.7810	0.0001	****
	Stage I–II vs Stage III–IV	–11.0859	0.4925	ns
**CD41a**	Control vs Stage I–II	–24.4561	0.0251	*
	Control vs Stage III–IV	–29.6358	0.0007	***
	Stage I–II vs Stage III–IV	–5.1797	1.0000	ns
**CD51_CD61**	Control vs Stage I–II	–37.3483	0.0002	***
	Control vs Stage III–IV	–46.1061	< 0.0001	****
	Stage I–II vs Stage III–IV	–8.7578	0.8153	ns

Pairwise Dunn's test with tie correction and continuity correction (0.5), Bonferroni adjustment for six planned comparisons per marker (α_adj = 0.05/6 = 0.0083); p (Bonf. adj.), Bonferroni-corrected two-tailed p; significance levels: ns > 0.05; * ≤ 0.05; ** ≤ 0.01; * ≤ 0.001; **** ≤ 0.0001.**

Supplementary Table S8 – Global Kruskal–Wallis test for histopathological-grade comparisons

Variable	H (χ^2)	p	Significance
**EVs_Total_AnnexinV**	12.342	0.0063	**
**CD66**	38.465	< 0.0001	****
**CD16**	34.160	< 0.0001	****
**CD3**	24.874	< 0.0001	****
**CD14**	11.106	0.0112	*
**CD45**	24.068	< 0.0001	****
**CD235a**	17.594	0.0002	***
**CD41a**	13.875	0.0010	***
**CD51_CD61**	33.268	< 0.0001	****

H, Kruskal–Wallis χ^2 statistic; p, two-tailed p value; significance levels: ns > 0.05; * ≤ 0.05; ** ≤ 0.01; * ≤ 0.001; **** ≤ 0.0001.**

Supplementary Table S9. Comprehensive pairwise Dunn's post hoc comparisons of circulating extracellular vesicle markers across control and histopathological grade subgroups

Variable	Comparison	Mean rank diff	p (Bonf. adj.)	Significance
**EVs_Total_AnnexinV**	Control vs I	–28.37	0.2506	ns
	Control vs II	–15.15	0.5228	ns
	Control vs III	–28.63	0.0044	**
	I vs II	13.22	1.0000	ns
	I vs III	–0.26	1.0000	ns
	II vs III	–13.48	0.5362	ns
**CD66**	Control vs I	–56.32	0.0003	***
	Control vs II	–45.14	< 0.0001	****
	Control vs III	–46.22	< 0.0001	****
	I vs II	11.18	1.0000	ns
	I vs III	10.10	1.0000	ns
	II vs III	–1.08	1.0000	ns
**CD16**	Control vs I	–51.68	0.0013	**
	Control vs II	–37.34	0.0001	***
	Control vs III	–46.43	< 0.0001	****
	I vs II	14.33	1.0000	ns
	I vs III	5.25	1.0000	ns
	II vs III	–9.08	1.0000	ns
**CD3**	Control vs I	–36.23	0.0560	ns
	Control vs II	–30.04	0.0042	**
	Control vs III	–41.43	< 0.0001	****
	I vs II	6.19	1.0000	ns
	I vs III	–5.20	1.0000	ns
	II vs III	–11.39	0.9070	ns
**CD14**	Control vs I	–35.42	0.0662	ns
	Control vs II	–17.86	0.2627	ns
	Control vs III	–24.94	0.0196	*

	I vs II	17.56	1.0000	ns
	I vs III	10.48	1.0000	ns
	II vs III	–7.08	1.0000	ns
**CD45**	Control vs I	–24.17	0.4861	ns
	Control vs II	–36.07	0.0003	***
	Control vs III	–37.17	< 0.0001	****
	I vs II	–11.90	1.0000	ns
	I vs III	–13.00	1.0000	ns
	II vs III	–1.10	1.0000	ns
**CD235a**	Control vs I	–37.49	0.0429	*
	Control vs II	–24.81	0.0305	*
	Control vs III	–32.90	0.0006	***
	I vs II	12.68	1.0000	ns
	I vs III	4.59	1.0000	ns
	II vs III	–8.08	1.0000	ns
**CD41a**	Control vs I	–32.65	0.1151	ns
	Control vs II	–23.53	0.0475	*
	Control vs III	–30.58	0.0019	**
	I vs II	9.12	1.0000	ns
	I vs III	2.08	1.0000	ns
	II vs III	–7.04	1.0000	ns
**CD51_CD61**	Control vs I	–48.98	0.0026	**
	Control vs II	–38.77	< 0.0001	****
	Control vs III	–45.69	< 0.0001	****
	I vs II	10.21	1.0000	ns
	I vs III	3.29	1.0000	ns
	II vs III	–6.91	1.0000	ns

Pairwise Dunn's test with tie correction and continuity correction (0.5), Bonferroni adjustment for six planned comparisons per marker (α_adj = 0.05/6 = 0.0083); p (Bonf. adj.), Bonferroni-corrected two-tailed p; significance levels: ns > 0.05; * ≤ 0.05; ** ≤ 0.01; * ≤ 0.001; **** ≤ 0.0001.**
