# Supplementary material for: Elevated Circulating Extracellular Vesicles as Prognostic Biomarkers in Cervical Cancer Progression
Source: Biomedicines. 2026 Jun 30;14(7):1492. doi: 10.3390/biomedicines14071492 (PMC13404281; doi:10.3390/biomedicines14071492)
Supplement: Supplementary file 1 [file biomedicines-14-01492-s001.zip › biomedicines-3674133-Supplementary_Figures.pdf]

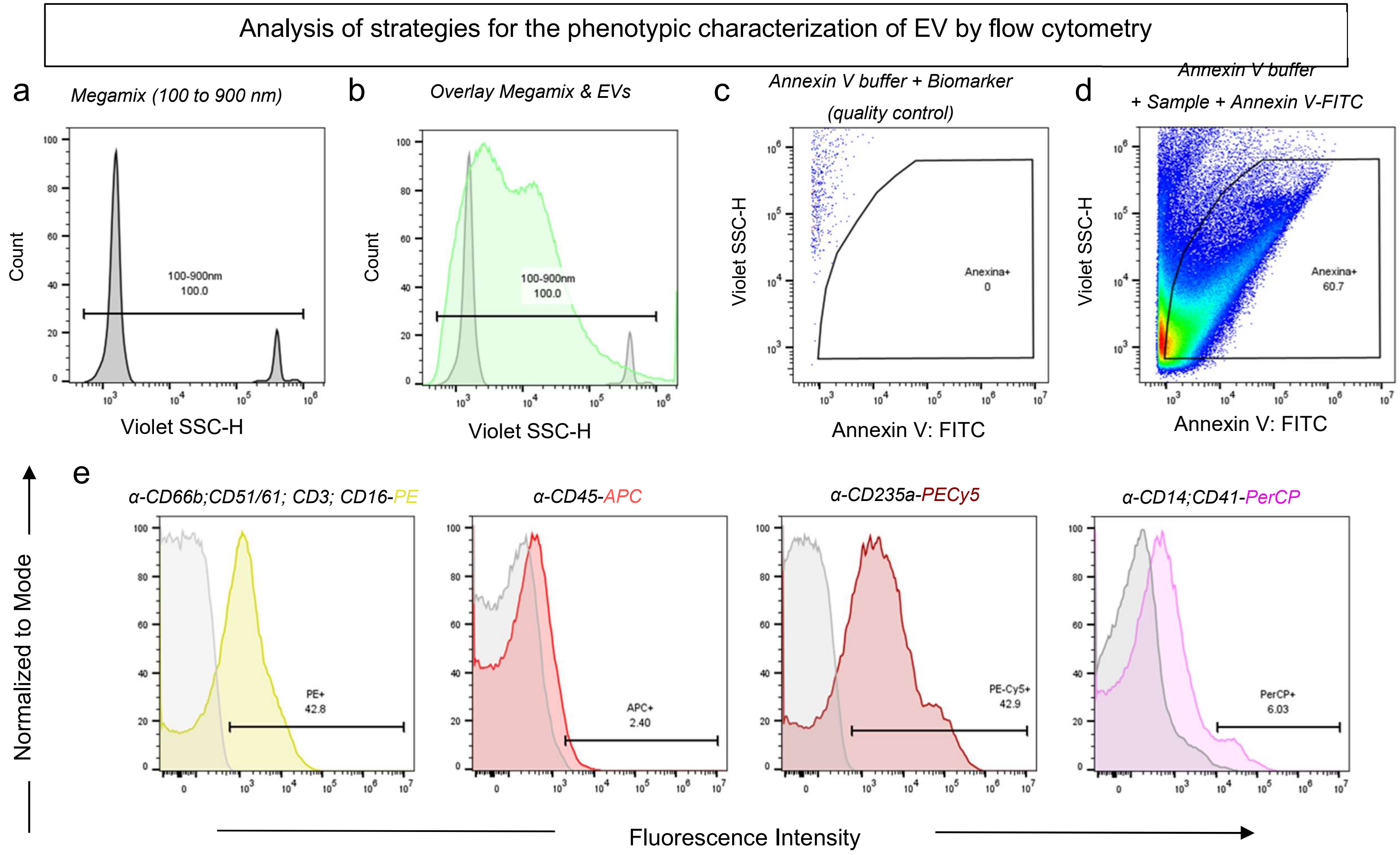

Analysis of strategies for the phenotypic characterization of EV by flow cytometry
a
Megamix (100 to 900 nm)
Count
100-900nm
100.0
Violet SSC-H
b
Overlay Megamix & EVs
Count
100-900nm
100.0
Violet SSC-H
c
Annexin V buffer + Biomarker
(quality control)
Violet SSC-H
Anexina+
0
Annexin V: FITC
d
Annexin V buffer
+ Sample + Annexin V-FITC
Violet SSC-H
Anexina+
60.7
Annexin V: FITC
e
α-CD66b;CD51/61; CD3; CD16-PE
PE+
42.8
α-CD45-APC
APC+
2.40
α-CD235a-PECy5
PE-Cy5+
42.9
α-CD14;CD41-PerCP
PerCP+
6.03
Normalized to Mode
Fluorescence Intensity

Figure S1. Representative strategy for the phenotypic characterization of extracellular vesicles (EV) by flow cytometry according to size range. a) The histogram shows two peaks of different sizes of beads, representing 100 nm and 900 nm, respectively. b) Histogram representing an sample (green) overlayed on different sizes of beads (100 nm and 900nm, respectively). c) Two-dimensional plot of Annexin V-FITC *vs* Violet SSC-H with EV point distribution of size between 100 and 900 nm in the control tube (Annexin V buffer + Sample) allows positioning of the positivity region for Annexin V-FITC with a lower threshold at 4.5%. This selected region is applied to all immunophenotyping tubes. d) One-way fluorescence intensity histograms are used for the quantification of phenotype-specific EV in percentage using the control tube (gray curves) to position the positivity threshold marker (color curves). The results are expressed in EV number/mm^3, considering the aspirated volume in 2 min of acquisition. e) Superimposed histograms of the fluorescences used, from negative and positive samples, which demonstrate the positioning of the gate after the negative (gray) peak, with the part of the colored peak that enters the gate being the positivity for that fluorescence.
