# Supplementary material for: Elevated Circulating Extracellular Vesicles as Prognostic Biomarkers in Cervical Cancer Progression
Source: Biomedicines. 2026 Jun 30;14(7):1492. doi: 10.3390/biomedicines14071492 (PMC13404281; doi:10.3390/biomedicines14071492)

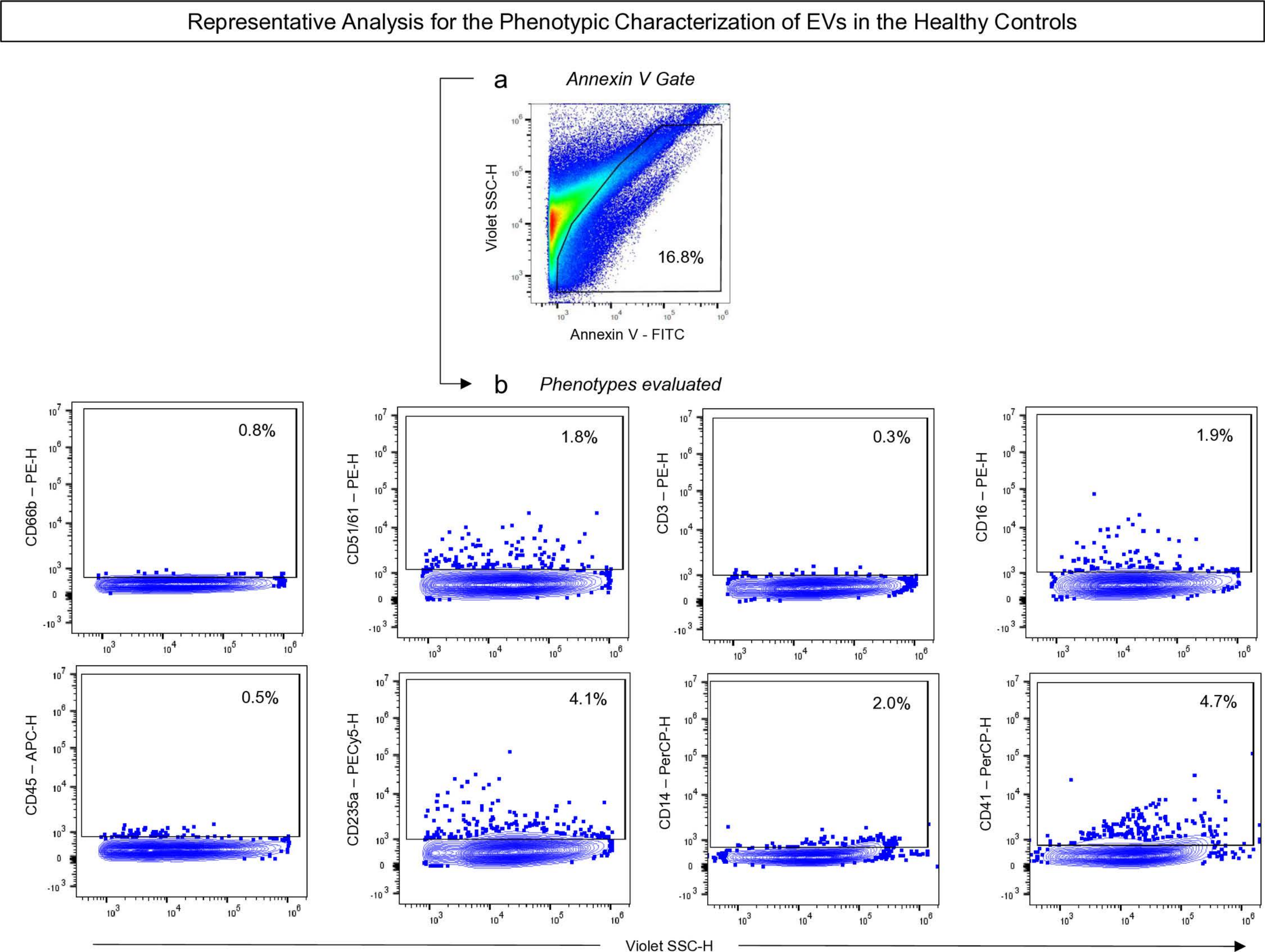

Representative Analysis for the Phenotypic Characterization of EVs in the Healthy Controls
a
Annexin V Gate
Violet SSC-H
Annexin V - FITC
16.8%
b
Phenotypes evaluated
CD66b – PE-H
0.8%
CD51/61 – PE-H
1.8%
CD3 – PE-H
0.3%
CD16 – PE-H
1.9%
CD45 – APC-H
0.5%
CD235a – PECy5-H
4.1%
CD14 – PerCP-H
2.0%
CD41 – PerCP-H
4.7%
Violet SSC-H

Representative Analysis for the Phenotypic Characterization of EVs in the Patient Cohort

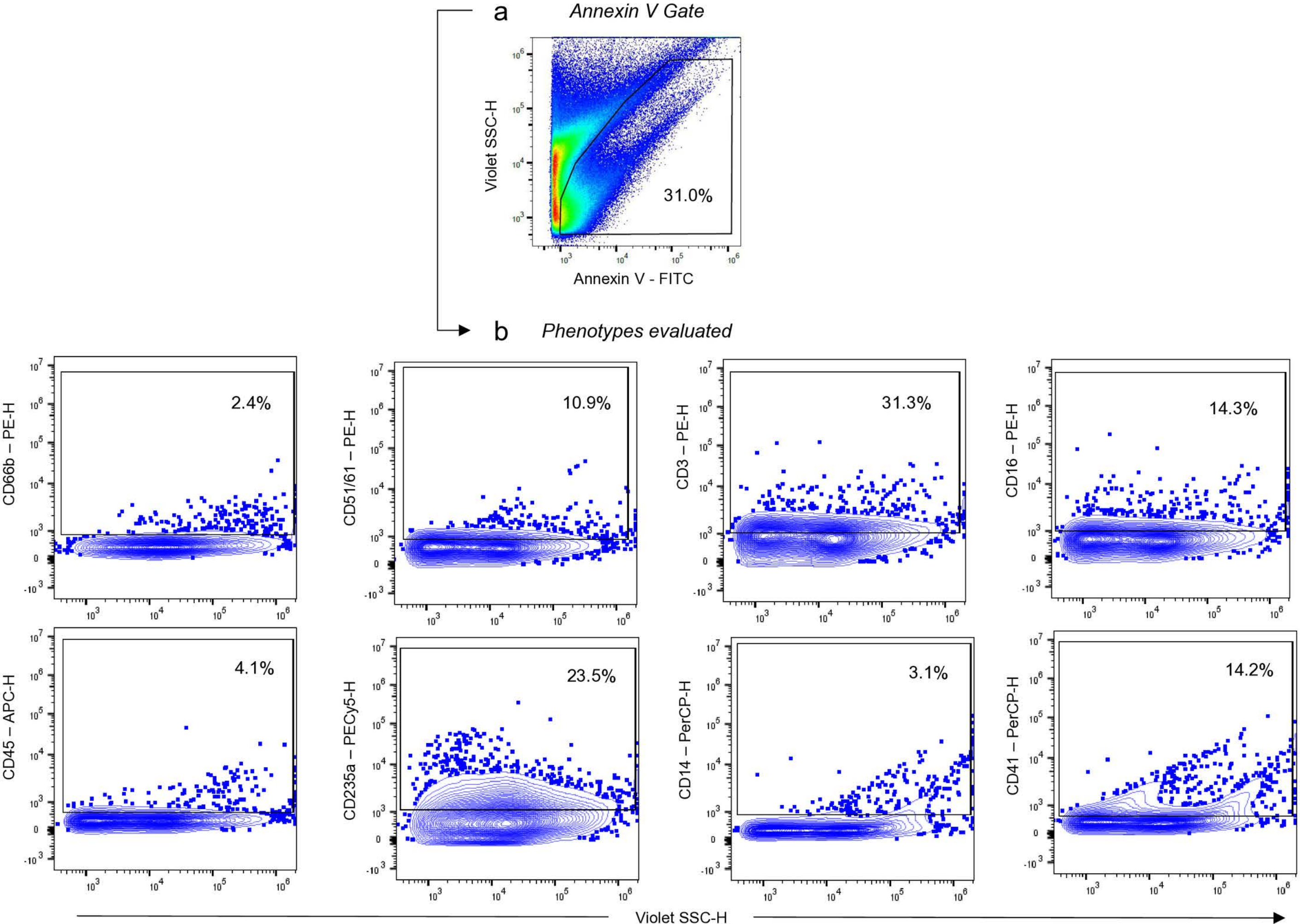

Supplement: Supplementary file 1 [file biomedicines-14-01492-s001.zip › Supplementary Figures S2 and S3.pdf]
