# Supplementary material for: Elevated Circulating Extracellular Vesicles as Prognostic Biomarkers in Cervical Cancer Progression
Source: Biomedicines. 2026 Jun 30;14(7):1492. doi: 10.3390/biomedicines14071492 (PMC13404281; doi:10.3390/biomedicines14071492)
Supplement: Supplementary file 1 [file biomedicines-14-01492-s001.zip › Legends for Supplementary Figuress 2 and 3docx.pdf]

Legends for Supplementary Figures

Figure S2. Representation of the gating strategy for the phenotypic characterization of extracellular vesicles (EVs) in the healthy controls. a) Representative Annexin V-FITC *vs*. Violet SSC-H dot plot illustrating the gating strategy for selecting Annexin V-positive EVs. The delimited region (Annexin V Gate) defines the EV population used for subsequent phenotypic analyses. b) Representative contour plots of the phenotypes evaluated in the cohort. Each plot displays the staining for a specific marker (Y-axes: CD66b, CD51/CD61, CD3, CD16, CD45, CD235a, CD14, and CD41) against the size/complexity parameter Violet SSC-H (X-axis). The upper rectangular gates indicate the percentage of EVs positive for Annexin V in each evaluated phenotype.

Figure S3. Representation of the gating strategy for the phenotypic characterization of extracellular vesicles (EVs) in the patient cohort. a) Representative Annexin V-FITC vs. Violet SSC-H density plot illustrating the gating strategy for selecting Annexin V-positive EVs. The delimited region (Annexin V Gate) defines the EV population used for subsequent phenotypic analyses. b) Representative contour plots of the phenotypes evaluated in the cohort. Each plot displays the staining for a specific marker (Y-axes: CD66b, CD51/CD61, CD3, CD16, CD45, CD235a, CD14, and CD41) against the size/complexity parameter Violet SSC-H (X-axis). The upper rectangular gates indicate the percentage of EVs positive for Annexin V in each evaluated phenotype.
